# Compound Eye Adaptations for Diurnal and Nocturnal Lifestyle in the Intertidal Ant, *Polyrhachis sokolova*

**DOI:** 10.1371/journal.pone.0076015

**Published:** 2013-10-14

**Authors:** Ajay Narendra, Ali Alkaladi, Chloé A. Raderschall, Simon K. A. Robson, Willi A. Ribi

**Affiliations:** 1 ARC Centre of Excellence in Vision Science, Research School of Biology, The Australian National University, Canberra, Australian Capital Territory, Australia; 2 Department of Biological Sciences, Faculty of Science, King Abdulaziz University, North Campus, Jeddah, Saudi Arabia; 3 Centre for Tropical Biodiversity & Climate Change, School of Marine and Tropical Biology, Faculty of Science and Engineering, James Cook University, Townsville, Queensland, Australia; 4 Private University of Liechtenstein, Triesen, Principality of Liechtenstein; University of Sussex, United Kingdom

## Abstract

The Australian intertidal ant, *Polyrhachis sokolova* lives in mudflat habitats and nests at the base of mangroves. They are solitary foraging ants that rely on visual cues. The ants are active during low tides at both day and night and thus experience a wide range of light intensities. We here ask the extent to which the compound eyes of *P. sokolova* reflect the fact that they operate during both day and night. The ants have typical apposition compound eyes with 596 ommatidia per eye and an interommatidial angle of 6.0°. We find the ants have developed large lenses (33 µm in diameter) and wide rhabdoms (5 µm in diameter) to make their eyes highly sensitive to low light conditions. To be active at bright light conditions, the ants have developed an extreme pupillary mechanism during which the primary pigment cells constrict the crystalline cone to form a narrow tract of 0.5 µm wide and 16 µm long. This pupillary mechanism protects the photoreceptors from bright light, making the eyes less sensitive during the day. The dorsal rim area of their compound eye has specialised photoreceptors that could aid in detecting the orientation of the pattern of polarised skylight, which would assist the animals to determine compass directions required while navigating between nest and food sources.

## Introduction

Ants are one of the most dominant insects to have colonised a range of ecological and temporal niches. Within these different niches they cope with dramatic variation in ambient light intensity. Light intensity at night is a million times dimmer than in the day [1] and nocturnal insects have evolved an array of visual adaptations to forage at these low light levels. In comparison to diurnal ants, nocturnal ants typically have larger lenses (night-active *Myrmecia pyriformis*: 38 µm [2]; day-active *Melophorus bagoti*: 19 µm [3]) and wider rhabdoms (night-active *M. pyriformis*: 6 µm [2]; day-active *M. bagoti*: 1.6 µm [3]) to increase photon capture rate in dim-lit conditions. Such visual adaptations are also seen in bees (diurnal: *Apis mellifera*; nocturnal: *Megalopta genalis* [4]–[6]) and wasps (diurnal: *Polistes occidentalis*; nocturnal: *Apoica pallens* [7]). This increase in the size of the lens and the rhabdom diameter is independent of body size and occurs even within congeneric species that are active at different times of the day (*Myrmecia* ants [2], *Xylocopa* bees [6]). Variation in the compound eye structure of ants is also dependent on their style of locomotion (flying/pedestrian), thus leading to striking differences of the visual system even within a single species [8]. Nocturnal insects in addition employ neural mechanisms to spatially and temporally pool photoreceptor signals to increase the signal-to-noise ratio [9]–[12].

**Figure 1:**
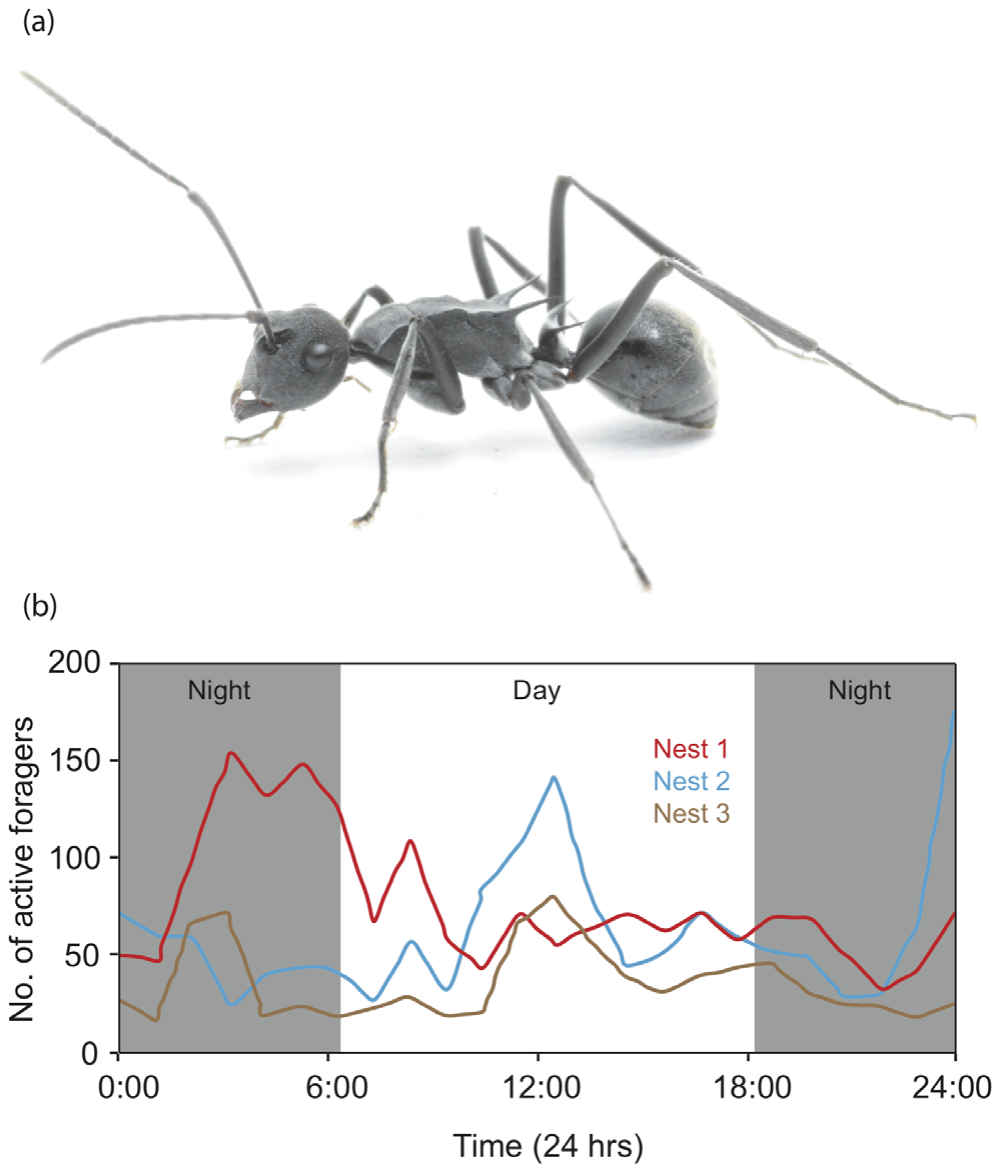
The intertidal ant *Polyrhachis sokolova* and its typical activity. (a) A worker of *P. sokolova*. (b) Daily activity schedule of ants from three nests determined by counts of outgoing and returning foragers in 5-minute bins over a 24-hr period on a single day in the month of April. This illustrates that ants are active during both day and night.

The Old World ant genus *Polyrhachis* is represented by nearly 500 species [13]. With their long spinous structures and remarkable colour variation they are arguably one of the most morphologically diverse group of ants. These ants occupy a variety of micro-niches ranging from subterranean to terrestrial, while others are arboreal or nest within wood [13]. Very little is known about their foraging behavior, other than a few observations provided in taxonomic literature. From this we know that several species of *Polyrhachis* forage individually, some resort to carrying nestmates and very few in fact establish and follow pheromone trails. In addition, some species are strictly diurnal while others are nocturnal. Here we provide the first description of the compound eye structure for the ant genus *Polyrhachis* by studying the intertidal ant, *Polyrhachis sokolova* (Fig. 1a). These ants are unique in establishing nests in the mangrove habitats at the ocean and land interface [14]. The ants are active during low tides, at both day and night (Fig. 1b), with most animals returning to the nest before the water level rises. They thus experience a wide range of light intensities. Ants active at both bright and dim light intensities need to navigate to specific locations. Ants typically derive compass information from celestial cues such as the pattern of polarised skylight [15], [16]. When available, ants use landmark information to determine heading directions and to pinpoint locations of food source and nest [17]–[20]. Workers of *P. sokolova* are individually foraging ants that use both landmark information and celestial cues to determine heading direction [21]. The pattern of polarised skylight is detected through a specialised set of photoreceptors in the dorsal rim area (DRA) of the compound eye [22], [23]. In the DRA of ants, the rhabdoms are dumbbell-shaped with two sets of retinula cells contributing microvilli with orthogonal orientation to each other. The absorption of linearly polarised light is maximal when the e-vector is parallel to the long axis of the microvilli. Both day- and night-active ants have been shown to respond to a change in the orientation of polarised skylight [16], [24]. The DRA has been documented in several day-active [23], [25], and one night-active ant species [26]. Both diurnal and nocturnal ants also rely on landmark information for navigation [16], [19], [27]–[29], which requires sufficient resolution and sensitivity. In addition, eyes that must work in such wide range of light conditions must have some means to adjust their sensitivity. Insects with apposition eyes often control light flux to the photoreceptors through a migration of primary pigments around the crystalline cone and radial migration of screening pigments within the retinula cells surrounding the rhabdom [30]–[33]. Here we ask to what degree the compound eyes of *P. sokolova* reflect the fact that these ants are able to operate at both day and night.

## Methods

### Study species

We collected workers of the solitary foraging intertidal ant *Polyrhachis sokolova* (Fig. 1a) from two nests in the mangrove habitats of Pallarenda (19°12′32.76″S, 146°46′26.82″E), Townsville, QLD, Australia. The ants are monomorphic and exhibit very little variation in body size (body length: 10.82±0.12 mm; head width: 1.86±0.10 mm; n = 5). The ants are found along the Australian east coast from Torres Strait to Gladstone in Queensland and also in neighbouring tropical countries [34], [35].

*Ethics statement*: Ants were studied under Queensland Government Department of National Parks, Recreation, Sport & Racing Permit ATH 12/011. Animal Ethics approval to study ants is required under Federal, State or institution (James Cook University) Guidelines. Field studies did not involve endangered or protected species.

### Analysis of the visual system

#### Facet numbers, size and distribution

We covered the compound eyes with a thin layer of colourless nail polish to produce cornea replicas [8], [36]. Once dry, the replicas were carefully removed and flattened on a microscope slide by making incisions with a micro-scalpel. The replicas were photographed in a Zeiss light microscope. We determined facet numbers and facet diameters of five individuals. We used one replica to map the facet area and distribution using a custom-written program in Matlab (© Richard Peters, La Trobe University).

#### Histology

To identify light- and dark-adaptation mechanisms, we fixed their eyes under natural light conditions at 10 am (light-adapted) and at 10 pm (dark-adapted). In both cases, live animals were kept in transparent glass jars and placed outdoors to experience ambient light intensities for 24 hrs before dissection. Ants were immobilised on ice, their mandibles removed and head capsules opened. Optimal retinal fixation was achieved by cutting the most ventral rim of the eye. Specimens were fixed for four hours in a mixture of 2.5% glutaraldehyde and 4% paraformaldehyde in phosphate buffer (pH 7.2 – 7.5). This was followed by a series of buffer washes and post-fixation in 2% OsO_4_ in distilled water for one hour. Samples were then dehydrated in an ethanol series, transferred to propylene oxide and embedded in Epoxy resin (FLUKA). One-micron thick cross-sections of ommatidia from the frontal region of the eye and of the dorsal region of the eye were cut on a Reichert Ultracut microtome using glass or diamond knives. Sections for light microscopy were stained with toluidine blue and digitally photographed in a Zeiss photo-microscope, while ultra-thin sections for transmission electron microscopy were stained with 6% saturated uranyl acetate (25 min) and lead citrate (5 min) before viewing with a Hitachi transmission electron microscope.

#### Theoretical calculations

(a) Optical sensitivity, *S,* defines the light-gathering capacity of an eye [37], [38]: 
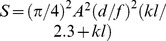
, where *A* = largest facet diameter (µm); *d* = diameter of the rhabdom (µm); f =  focal length, determined by the distance from the centre of curvature of the inner corneal lens surface (as an estimate for the position of the nodal point) to the tip of the rhabdom; *l* = the rhabdom length; *k = *absorption coefficient assumed to be 0.0067 µm^−1^. (b) Interommatidial angle, Δø, was determined by two methods: (i) assuming each eye has a hemispherical visual field and dividing it by the number of facets and (ii) using lens diameters and eye radius. (i) 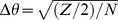, where, Z  =  Sphere  = 41252.96 square degrees; N = facet number ii) 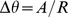, where *A* =  largest lens diameter *R* =  eye radius =  
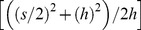
, which estimates the radius of the eye from the length of a segment (s) transecting the eye and its height (h) [3]. (c) Acceptance angle (Δ*ρ*), 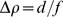, [39], [40], where d = diameter of rhabdom (µm); f = focal length (µm)

## Results

**Figure 2:**
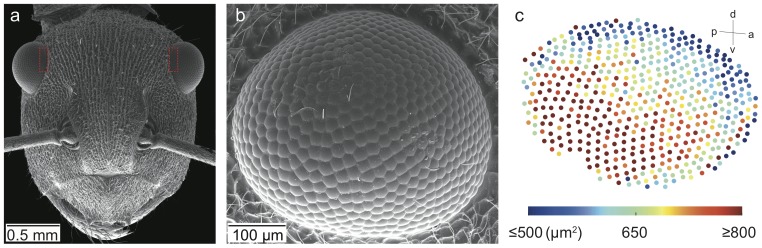
External morphology of the eye structure of *Polyrhachis sokolova*. Scanning electron micrographs (SEM) illustrate (a) a frontal view of the head with dorsal rim area indicated by a red dashed box; (b) a lateral view of the right eye; (c) an eye map indicating the facet size and facet distribution. Orientation of the eye (for b,c) is indicated in the top right: a: anterior, p: posterior, v: ventral; d: dorsal.

**Figure 3:**
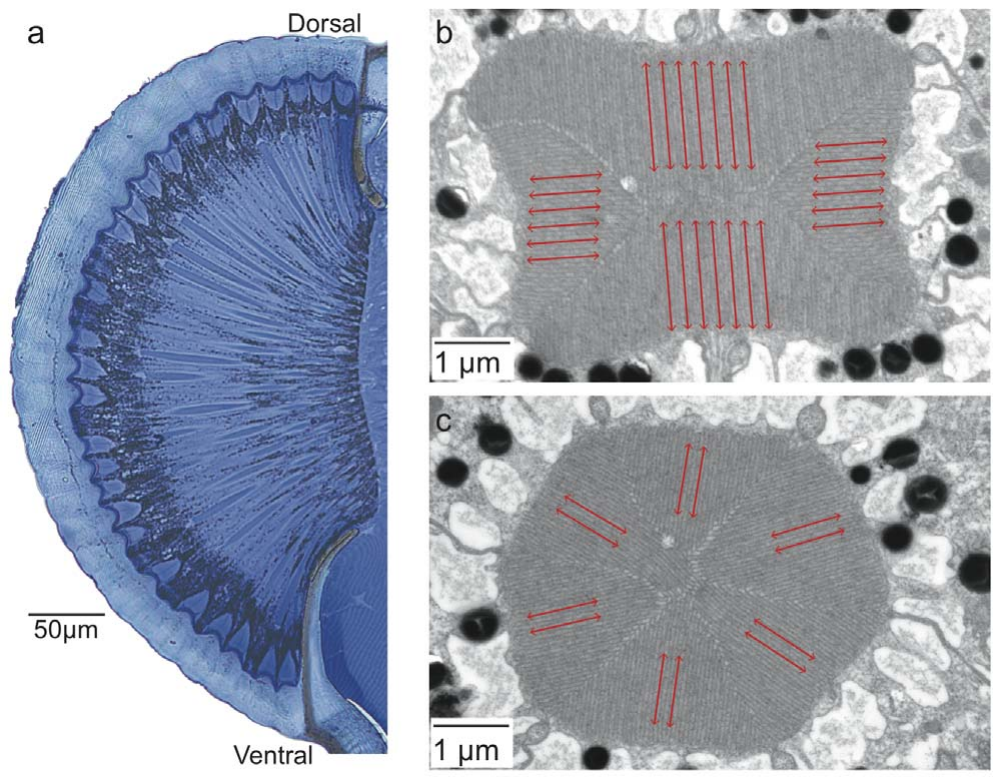
Histological analysis of the eye of *Polyrhachis sokolova*. (a) Frontal section of the eye from the dorsal to ventral region. Electron micrographs of the rhabdoms in the (b) dorsal rim area (DRA) of the eye, and (c) in the medio-frontal region. Microvilli orientation in the rhabdoms is indicated by red lines.

Similar to other ants, *P. sokolova* have a pair of compound eyes of the apposition type. Each compound eye has 596.2±51.7 (n = 5) facets (Fig. 2). They have an average facet diameter of 23.5±3.0 µm (mean ± s.d., n = 50, 5 animals), with the largest facets (33 µm) found in the posterior and ventral region of the eye (Fig. 2). In the frontal region of the eye the rhabdoms are hexagonal in shape and formed by eight retinula cells (six retinula cells are visible in the distal tip of the rhabdom, Fig. 3c), with microvilli arranged in three different orientations. The diameter of the rhabdom as measured from cross sections of the frontal region of the eye was 5.0±0.2 µm (n = 20, 5 animals). The length of the rhabdom varied from 79 µm (dorsal) to 135 µm (frontal) to 113 µm (ventral) region. A distinct dorsal rim area (DRA) was present for four rows of ommatidia. In the DRA, the rhabdoms (each formed by eight receptor cells) were rectangular within which the microvilli were oriented in only two orthogonal directions, i.e., microvilli were oriented 90° towards each other (Fig. 3b). Based on these measures, we determined the average interommatidial angle (Δ*θ*) to be 5.9° (assumption of eye having a hemispherical visual field) or 6.0° (determined as Δ*θ* = A/R) and the optical sensitivity *S* of the eye to be 1.15 µm^2^sr.

**Figure 4:**
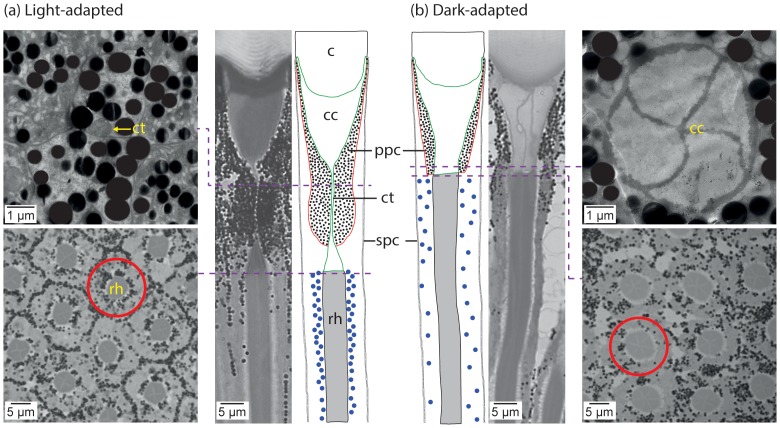
Pupillary mechanism in *Polyrhachis sokolova*. (a) Light- and (b) dark- adaptation in the compound eye. Transverse sections through the crystalline cone tract (top left) and crystalline cone (top right); transverse sections through the rhabdom (bottom left and bottom right); longitudinal sections and illustration of a single ommatidium. Red circle indicates the retinula cell screening pigments close to the rhabdom in the light-adapted state, but farther from the rhabdom in the dark-adapted state. c – cornea; cc – crystalline cone; ct – crystalli ne cone tract; ppc – primary pigment cells; spc – secondary pigment cells; rh – rhabdom. Dashed line indicates the sectioning depth. Filled blue circles in longitudinal illustrations – retinula cell pigments.

In the light-adapted eye the primary pigment cells constrict the crystalline cone to form a narrow tract less than 0.5 µm wide and 16 µm long (Fig. 4a). The formation of the narrow crystalline cone tract doubled the distance between the crystalline cone and the distal tip of the rhabdom. The diameter of the distal rhabdom in the light-adapted eye was 5 µm, the focal length was 64.32 µm, resulting in an acceptance angle of 4.45°. The retinula cell pigments tightly ensheathed the rhabdom in the light-adapted eye. In the dark-adapted eye, the primary pigment pupil opened up to 4.8 µm wide aperture (Fig. 4b). The focal length measured 40.53 µm and the acceptance angle was 8.48°. In the dark-adapted eye the rhabdom diameter increased to 6 µm and the retinula cell pigments migrated away from the rhabdom.

## Discussion

The apposition compound eyes of *P. sokolova* are equipped with large lenses and wide rhabdoms that are typical optical adaptations for low light conditions. To protect their sensitive eyes in bright light conditions, they have developed light adaptation mechanisms that include a primary pigment pupil that constricts the light path to the rhabdom to a 0.5 µm narrow aperture and the radial movement of retinula screening pigments close to the rhabdom. These ants posses a distinct dorsal rim area with specialised photoreceptors that most likely aid in detecting the pattern of polarised skylight.

Compared to the exclusively day-active ants, worker ants of *P. sokolova* have slightly more ommatidia per eye (590) than *M. bagoti* (499) [3], but significantly less than *Cataglyphis bicolor* (1300) [41] and *Myrmecia croslandi* (2363 facets) [2], which are all ants of similar body length. Workers of *P. sokolova* have larger facets (33 µm) than diurnal ants of comparable body size (*M. bagoti*: 19 µm; *C. bicolor*: 24 µm; *M. croslandi*: 22 µm) and are comparable to that found in the much larger nocturnal bull ant *M. pyriformis* (37 µm; body length: 27mm). Facet size in ants thus does not scale with body size but reflects the light levels at which animals are active. However, in *Camponotus* species, Menzi [42] discovered that facet sizes of nocturnal and diurnal ants were similar (ranging from 20–24 µm). It is possible that these nocturnal ants have evolved neural adaptations to capture light at low light levels. But we suspect that the nocturnal species investigated, *C. ligniperda* and *C. irritans*, show less reliance on visual information for navigation and rely more on chemical cues. In *P. sokolova*, the largest facets are located in the posterior-ventral region of the eye, which could be an indication of a region with increased sensitivity or better acuity. In the visually guided *Myrmecia* ants, the largest facets are typically found in the medio-frontal region of the eye and have been suggested to be a ‘bright-zone’ with increased sensitivity rather than being an ‘acute zone’ having better acuity [43]. We suspect this to be the case in *P. sokolova* too, which could be verified by measuring the interommatidial angles in different regions of the eye. In addition to having large facets, *P. sokolova* ants have large rhabdoms (5.0 µm) which are typical of nocturnal ants [2] and other nocturnal insects [4], [6]. Large rhabdoms increase the number of photons that can be captured, making the eye more sensitive to dim light conditions.

The average interommatidial angle determined by the two methods provided comparable outcomes of 5.9° and 6.0°, which is quite similar to the diurnal desert ants, *C. bicolor* (3.0°–5.0°) [44] and *M. bagoti* (3.0°–6.4°) [3]. The interommatidial angle of the nocturnal *M. pyriformis* varies from 1.1° in the lateral to 3.0° in the frontal region of the eye [43]. Ideal sampling can be inferred if the ratio of the acceptance angle (Δ*ρ*) and the interommatidial angle (ΔØ) is 2 [45] and this is the case in *M. pyriformis* [43]. In *P. sokolova*, this ratio ranges from 0.73 (light-adapted) to 1.21 (dark-adapted) indicating that ants under-sample the image in both states, but acquire enough contrast information for landmark guidance. Similar under-sampling is seen in *M. bagoti* [3], which also relies on landmark information for homing [46]. Determining the viewing directions of each ommatidium by the *in vivo* pseudopupil method would provide more accurate measures of the sampling resolution across the visual field, which among ants has been addressed in *Cataglyphis* [44].

Foragers of *P. sokolova* are active at both day and night. We found that ants have a distinct ‘pupil’ mechanism, by which they protect the photoreceptors from bright light. In bright light, the primary pigment cells constrict the crystalline cone to a narrow 0.5 µm tract, thus reducing the amount of light that reaches the photoreceptors. The acceptance angle of the rhabdom in a light-adapted eye reduced to 4.45°C ompared to 8.48° in a dark-adapted eye, thus decreasing the sensitivity of the eye. Very little light can travel through a 0.5 µm narrow aperture. The acceptance angle calculated for this narrow aperture, which has a focal length of 40.53 µm, reduces further to 0.70°. In the light-adapted eye, the distal tip of the rhabdom lies nearly twice the distance from the crystalline cone (23 µm more than in the dark-adapted eye), increasing the focal length, thereby further decreasing the sensitivity. Interestingly, the distal tip of the narrow crystalline cone tract in the light-adapted eye was positioned at the same distance from the lens as the distal tip of the rhabdom in the dark-adapted state (compare longitudinal sections in Figs. 4a, 4b). A constriction of the crystalline cone to form a narrow tract in response to an increase in light intensity occurs in other nocturnal ants such as *C. ligniperda*, *C. irritans* [42] and *M. pyriformis* [43] and in several other insects [30]–[33], [47], [48], but does not occur in strictly day-active ants. In day-active ants (*C. bicolor, Formica polyctena, Myrmecia gulosa*), the only light adaptation mechanism that has been observed is the radial migration of retinula cell screening pigments wherein the pigments tightly ensheath the rhabdom in the light-adapted state and move away from the rhabdom in the dark-adapted state [41], [49], [50]. In nocturnal ants, this radial migration of retinula screening pigment has been observed in *Camponotus* ants [42]. The extreme ‘pupil’ mechanism involving the constriction of the crystalline cone to a narrow tract by the primary pigment cells thus allows *P. sokolova* to be active in a range of light intensities.

The diameter of the distal rhabdom increased from 5 µm in the light-adapted state to 6 µm in the dark-adapted state. Such increase in rhabdom diameters has also been reported in the apposition eyes of crabs [51], [52] and locusts [53]. In the locusts, the area of the rhabdom increased from 3.6 µm in light-adapted state to 17.0 µm in the dark-adapted, a 4.7 fold increase. This is achieved by rapidly assembling new microvillar membrane in the dark-adapted state and shedding this membrane in the light-adapted state [53].

Foragers of *P. sokolova* derive compass information from both the landmark panorama and from celestial cues [21]. As a celestial cue, they most likely derive compass information from the pattern of polarised skylight most likely detecting it from the specialised photoreceptors in the DRA (Fig. 3b). In the DRA of *P. sokolova*, the rhabdoms are rectangular shaped with microvilli oriented in only two orthogonal directions similar to the DRA in ten other ant genera [22], [23], [25].

In summary, the intertidal ant *P. sokolova* is active during both diurnal and nocturnal low tides, thus experiencing a wide range of different light intensities. To cope with life at night, the ants have developed night-vision equipment, large lenses and wide rhabdoms. To protect their sensitive rhabdoms they posses a pupillary mechanism to restrict the light flux to the photoreceptors in bright light. In addition, they have a specialised set of photoreceptors in the DRA that could allow them to detect the orientation of the pattern of polarised skylight.
