# Correction: Compound Eye Adaptations for Diurnal and Nocturnal Lifestyle in the Intertidal Ant, *Polyrhachis sokolova*

**DOI:** 10.1371/annotation/b18fcad6-597e-4ba9-86bc-f21030ed3a1b

**Published:** 2013-11-12

**Authors:** Ajay Narendra, Ali Alkaladi, Chloé A. Raderschall, Simon K. A. Robson, Willi A. Ribi

References 21, 51, 52, and 53 are the incorrect references. The corrected references are as follows:

21. Narendra A, Raderschall CA, Robson SKA (2013) Homing abilities of the Australian intertidal ant Polyrhachis sokolova. Journal of Experimental Biology: 216, 3674-3681. doi:10.1242/jeb.089649

51. Leggett LMW, Stavenga DG (1981) Diurnal changes in angular sensitivity of crab photoreceptors. Journal of Comparative Physiology 144: 99-109.

52. Nässel DR, Waterman TH (1979) Massive diurnally modulated photoreceptor membrane turnover in crab light and dark-adaptation. Journal of Comparative Physiology 131: 205-216.

53. Williams DS (1982) Ommatidial structure in relation to turnover of photoreceptor membrane in the locust. Cell and Tissue Research 225: 595-617. 

